# Advances in Fatigue Performance of Metal Materials with Additive Manufacturing Based on Crystal Plasticity: A Comprehensive Review

**DOI:** 10.3390/ma17051019

**Published:** 2024-02-22

**Authors:** Wei Zhang, Anheng Wang, Jianbin Wang, Qiaoyu Wang, Fan Li, Kuai Lu

**Affiliations:** 1 School of Mechanical Engineering, Anhui Polytechnic University, Wuhu 241000, China; zhangwei202109@163.com (W.Z.); wjb@ahpu.edu.cn (J.W.); wangqiaoqu@163.com (Q.W.); lifan971213@163.com (F.L.); 2 Update Industry (Wuhu) Co., Ltd., Wuhu 241000, China; 006@updateparts.com

**Keywords:** metal additive manufacturing, crystal plasticity finite element, fatigue life prediction

## Abstract

Using metal additive manufacturing processes can make up for traditional forging technologies when forming complex-shaped parts. At the same time, metal additive manufacturing has a fast forming speed and excellent manufacturing flexibility, so it is widely used in the aerospace industry and other fields. The fatigue strength of metal additive manufacturing is related to the microstructure of the epitaxially grown columnar grains and crystallographic texture. The crystal plasticity finite element method is widely used in the numerical simulation of the microstructure and macro-mechanical response of materials, which provides a strengthening and toughening treatment and can reveal the inner rules of material deformation. This paper briefly introduces common metal additive manufacturing processes. In terms of additive manufacturing fatigue, crystal plasticity simulations are summarized and discussed with regard to several important influencing factors, such as the microstructure, defects, surface quality, and residual stress.

## 1. Introduction

Metal additive manufacturing is often used in the aerospace industry, medical equipment, weaponry, rail transit, and many other applications [1,2]. In the aerospace industry, the main load-bearing components, such as aircraft fuselage, frames, wing, landing gear, and key engine components, are manufactured by forging [3,4,5]. Forge pieces possess excellent comprehensive mechanical properties, but it is difficult to manufacture complex-shaped parts. Additive manufacturing using two-dimensional stacking to form three-dimensional entities of the processing principle can form complex-shaped mechanical parts [6,7]. This process makes up for traditional forging processes when manufacturing complex-shaped parts and is widely used. However, metal additive manufacturing still requires further improvement relative to the fatigue performance of forged components [8].

The key factors [9,10,11] affecting the fatigue performance of metal additive manufacturing mainly include the following. First is the effect of microstructures on the fatigue properties. Due to the influence of temperature gradients, the grain morphology of the primary phase in metal additive manufacturing is columnar grains that exhibit epitaxial growth. This inhomogeneous microstructure causes anisotropy in the macro-mechanical properties of the additive manufacturing. Second is manufacturing defects (pore, lack of fusion, etc.) that cause fatigue crack growth and reduce the service life of parts. Third is the problem of fatigue cracking induced by residual stress. Microscopic notches in rough surfaces can easily lead to fatigue crack initiation. From the essential relationship of “manufacturing process-material microstructure-fatigue performance,” the damage mechanism of fatigue in additive manufacturing is studied to establish a theoretical basis for optimizing process parameters and improving heat treatment schedules. Highly controllable fatigue performance is achieved by further improving the microstructure and reducing the defect level.

The crystal plasticity finite element method [12] is widely used in fields such as mechanics, material science, and mechanical research. Multi-scale numerical simulations based on crystal plasticity frameworks have gained widespread attention. The crystal plasticity finite element method can simulate various macroscopic mechanical responses of materials and characterize interactions between macroscopic boundary conditions and material microstructure evolution [13]. Crystal plasticity simulations show the strong advantage of multi-scale numerical simulations in exploring the more essential relationship between the “manufacturing process-material microstructure-fatigue performance” of metal additive manufacturing [14,15]. Many of the metal components in aerospace, weaponry, rail transit, etc., experience fatigue failure due to cyclic loading. In order to investigate the fatigue damage mechanism in metal additive manufacturing, this paper briefly reviews crystal plasticity simulations for the fatigue performance of metal additive manufacturing from crystal plasticity theory, with the aim of establishing a theoretical basis for improving the fatigue performance of metal additive manufacturing.

## 2. Metal Additive Manufacturing Formation Process

Additive manufacturing metal materials include titanium alloys [16,17], aluminum alloys [18,19], high-temperature alloys [20,21,22,23], steel [24,25,26], and magnesium alloys [27,28]. The raw material forms used in metal additive manufacturing include metal powders and wires [29], the most widely used of which is metal powder. The processing technology of metal additive manufacturing can be divided into two categories [30,31,32]: powder bed fusion (PBF) and direct energy deposition (DED). PBF featuring powder laying is an additive manufacturing process utilizing heat generated by lasers, electron beams, and infrared lamps to selectively melt/sinter raw materials in the area of the powder bed. The main representative technologies include selective laser melting (SLM) and electron selective beam melting (EBSM). DED featuring powder feeding is an additive manufacturing process that utilizes focused thermal energy to synchronously melt and deposit raw materials. Processing energy sources primarily include lasers, electron beams, and electric arcs. Common DED technologies include laser solid forming (LSF), electron beam free form fabrication (EBF^3^), and wire and arc additive manufacturing (WAAM). In addition, according to the different types of energy sources, metal additive manufacturing can be divided into laser additive manufacturing, electron beam additive manufacturing, and wire and arc additive manufacturing.

**Figure 1:**
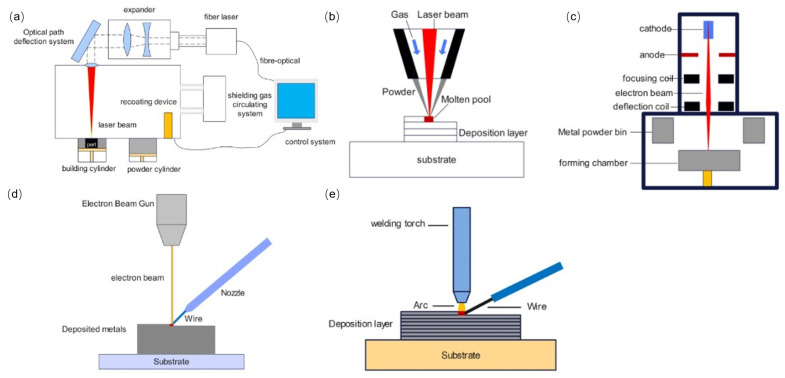
Schematic diagram of the working principle of metal additive manufacturing equipment. (**a**) SLM; (**b**) LSF; (**c**) EBSM; (**d**) EBF^3^; (**e**) WAAM.

SLM [33,34,35,36] is a commonly used metal additive manufacturing process. Its principle is to use laser beams as the energy source to selectively melt/sinter metal powder layer-by-layer based on two-dimensional information obtained by layering the part modeling data using software and ultimately manufacture the required three-dimensional solid parts (Figure 1a). Firstly, a horizontal scraper lays a layer of metal powder evenly on the substrate of the processing chamber; the laser beams selectively melt/sinter the metal powder based on the current two-dimensional profile information. The lifting system adjusts the substrate to descend, the horizontal scraper lays powder on the machined surface, and the equipment melts/sinters the current layer of metal powder. This cycle is repeated layer-by-layer until the manufacturing of the part is completed. The processing parameters significantly impact the quality of parts formed by SLM, making selecting the appropriate processing parameters necessary. The main processing process parameters include the power bed depth, scanning speed, laser power, and scanning interval [37,38,39,40]. The advantages of SLM are high dimensional accuracy, high surface quality, and excellent mechanical properties of the formed parts.

LSF [41,42,43] uses laser beams to melt the metal powder sprayed by the powder feeding nozzle into a liquid state, which is then stacked and solidified through a planned motion path to achieve a near-net-shape of the metal parts (Figure 1b).

EBSM [44,45,46,47] works similarly to SLM, using electron beams as the energy source for the additive manufacturing process. In a vacuum environment, the high-energy electron beam is controlled by a deflection coil to selectively melt/sinter metal powders along a planned path (Figure 1c).

EBF^3^ [48,49,50] uses high-energy electron beams as the heat source to melt/sinter the metal wire fed by the wire-feeding device. In a vacuum environment, equipment builds up point-by-point, line-by-line, and surface-by-surface until the metal part is formed (Figure 1d). Metal materials have lower reflectivities to electron beams than lasers, giving significant advantages of high energy density and high energy utilization. Electron beam additive manufacturing has the characteristics of high power and high radiation and needs to be carried out in a vacuum environment [51,52]. Thus, the equipment cost is high. Radiation leakage occurring during production can cause environmental pollution. Additionally, electron beams can only be used for processing conductive materials.

WAAM [53,54] uses high-energy arcs as energy sources and metal wires as the processed raw materials (Figure 1e). WAAM has the advantages of high efficiency, mature technology, and low cost. In addition, this process is more suitable for processing aluminum alloys.

## 3. Review of the Development of Crystal Plasticity Theory

The combination of crystal plasticity theory, including dislocation slip and finite elements, called the crystal plasticity finite element method, establishes a bridge between the macroscopic mechanical response and microscopic slip and shear in mesoscopic scales to characterize the fatigue damage process of materials. The early work of crystal plasticity theory can be traced back to the 1920s. In 1934, Taylor [55] believed on the basis of shear deformation that the essence of single-crystal plastic deformation was crystal shear based on dislocation slip and quantitatively described it. Later, Hill and Rice [56,57] established a crystal plasticity theoretical framework for analyzing single-crystal rate-independent behaviors. Subsequently, crystal plasticity theories for rate-dependent [58,59] and containing deformation twins [60,61,62,63,64,65] have been proposed. These studies have improved the crystal plasticity constitutive theory, and the crystal plasticity constitutive theory for large deformation is now basically complete. Regarding the crystal plasticity constitutive theory, Asaro [66] and Roters et al. [13] gave systematic presentations.

Regarding the constitutive theory of polycrystalline plasticity, the polycrystalline plastic response can be regarded as the common action of all single-crystal plastic responses. The transition theory that relates the mechanical response of a single crystal to multiple crystals is the homogenization method, and the representative homogenization method includes the Sachs polycrystalline model, Taylor model [67,68,69,70,71,72], and self-consistent model [73,74,75,76,77,78]. In terms of plastic-deformation mechanisms, the crystal plasticity framework is currently mainly used to predict the mechanical response induced by dislocation slip [79,80,81,82,83] and deformation twins. Some studies have also considered other special deformation modes, such as martensitic phase transition [84], shear banding [85,86], dislocation high-temperature climb [87,88], and creep [89]. The crystal plasticity constitutive theory includes the phenomenological crystal plasticity theory and the crystal plasticity theory based on physical mechanisms. The phenomenological crystal plasticity model is established by describing experimental phenomena. The crystal plasticity model based on physical mechanisms can explain the physical essence of plastic deformation [90] by considering the microstructure evolution [91,92] (such as dislocation density), and the mathematical model of crystal plasticity is established in the form of state variables [93]. On the meso-scale, crystal plasticity theory has become a hot research topic in the field of materials science because it more accurately characterizes the plastic deformation of metallic materials by using the flow model, the hardening model, and the internal state variable model. The rate-dependent crystal plasticity theory [94,95] can describe the relationship between material plastic deformation and loading path well. In terms of the interaction of slip systems, the rate-dependent crystal plasticity constitutive model introduces the self-hardening and latent hardening rate [96,97], which can be applied to metal materials based on dislocation slip deformation mechanisms. The following briefly introduces the single-crystal plastic constitutive theory for rate-dependent materials.

**Figure 2:**
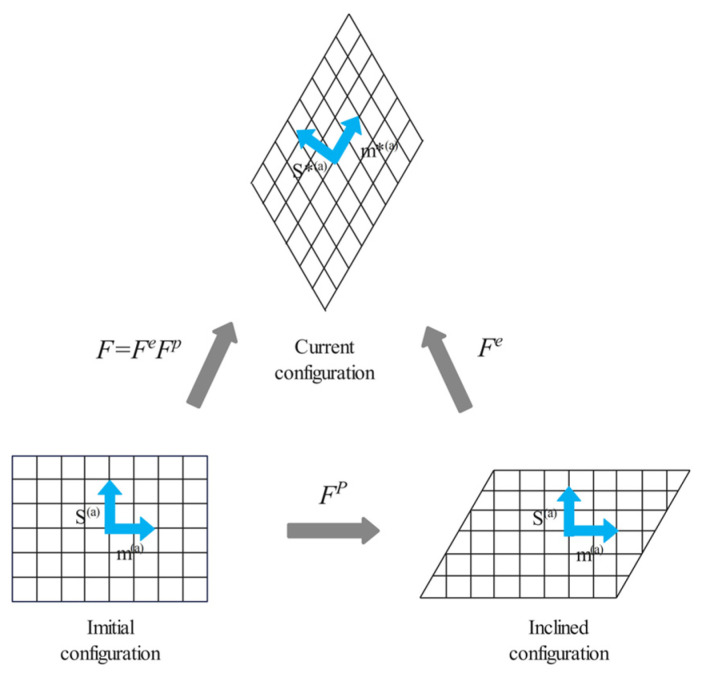
Decomposition of deformation.

In continuum mechanics, the deformation gradient *F* represents the deformation of an object. The theory of crystal plasticity suggests that crystal deformation comes from the plastic deformation part *F^p^* and the elastic deformation part *F^e^* (Figure 2), and the total deformation gradient *F* can be written as

(1) $$F=F^{e}F^{p}$$

Introduce the velocity gradient, denoted by *L*:

(2) $$L=\frac{\partial v}{\partial x}=\frac{\partial v}{\partial X}\frac{\partial X}{\partial x}=\dot{F}\cdot F^{-1}=L^{e}+L^{p}$$

where x represents the particle position of the current configuration, and *X* represents the particle position of the initial configuration.

Substituting (2) into (1) obtains

(3) $$L=L_{e}+L_{p}={\dot{F}}^{e}\cdot {\dot{F}}^{-1}+{\dot{F}}^{e}\cdot {\dot{F}}^{p}\cdot {\dot{F}}^{p-1}\cdot {\dot{F}}^{e-1}$$

where $L_{e}={\dot{F}}^{e}\cdot {\dot{F}}^{-1}$ and $L_{p}={\dot{F}}^{e}\cdot {\dot{F}}^{p}\cdot {\dot{F}}^{p-1}\cdot {\dot{F}}^{e-1}$.

Any n-order matrix can be expressed as the sum of a symmetric matrix and an antisymmetric matrix. Decompose the velocity gradient tensor into the deformation rate tensor *D* and the spin tensor *W* representing rotation:

(4) L=D+W

(5) $$D=\frac{1}{2}(L+L^{T})=D^{e}+D^{p}$$

(6) $$W=\frac{1}{2}(L-L^{T})=W^{e}+W^{p}$$

In the *α*th slip system before deformation, the unit vector of the slip direction is denoted by *S^(α^*^)^, and the unit normal vector of the slip surface is denoted by *m^(α)^*. As shown in Figure 2, the plastic deformation experiences shear only in the direction of slip and does not change the direction of *S^(α)^* and *m^(α)^*. Elastic deformation can cause the rotation and deformation of the crystal lattice, thus changing the magnitude and direction of *S^(α)^* and *m^(α)^*. After deformation, the sliding direction and the normal direction of the sliding surface are, respectively, represented as

(7) $$s^{{*}_{\left(\alpha \right)}}=F^{e}\cdot s^{{\;}_{\left(\alpha \right)}}\;m^{{*}_{\left(\alpha \right)}}=m^{{\;}_{\left(\alpha \right)}}\cdot F^{e-1}$$

One work [98] demonstrates that the plastic deformation velocity gradient in the intermediate configuration can be expressed as

(8) $${\dot{F}}^{p}\cdot F^{p-1}=\sum_{\alpha =1}^{N}{\dot{\gamma }}^{\left(\alpha \right)}s^{\left(\alpha \right)}⊗m^{\left(\alpha \right)}$$

where ${\dot{\gamma }}^{\left(\alpha \right)}$ denotes the shear strain rate caused by the *α*th slip system.

The plastic velocity gradient in the final configuration can be expressed as

(9) $$L^{p}=\sum_{\alpha =1}^{N}{\dot{\gamma }}^{\left(\alpha \right)}s^{*(\alpha )}⊗m^{*(\alpha )}$$

The plastic velocity gradient can also be expressed as

(10) $$L^{p}=D^{p}+W^{p}$$

(11) $$D^{p}=\frac{1}{2}\sum_{\alpha =1}^{N}(s^{*(\alpha )}⊗m^{*(\alpha )}+m^{*(\alpha )}⊗s^{*(\alpha )}){\dot{\gamma }}^{\left(\alpha \right)}$$

(12) $$W^{p}=\frac{1}{2}\sum_{\alpha =1}^{N}(s^{*(\alpha )}⊗m^{*(\alpha )}-m^{*(\alpha )}⊗s^{*(\alpha )}){\dot{\gamma }}^{\left(\alpha \right)}$$

Redefining the two formulas, the following emerge:

(13) $$P^{\left(\alpha \right)}=\frac{1}{2}(s^{*(\alpha )}⊗m^{*(\alpha )}+m^{*(\alpha )}⊗s^{*(\alpha )})$$

(14) $$W^{\left(\alpha \right)}=\frac{1}{2}(s^{*(\alpha )}⊗m^{*(\alpha )}-m^{*(\alpha )}⊗s^{*(\alpha )})$$

Then, there are

(15) $$D^{p}=\sum_{\alpha =1}^{N}P^{\left(\alpha \right)}{\dot{\gamma }}^{\left(\alpha \right)}\;W^{p}=\sum_{\alpha =1}^{N}W^{\left(\alpha \right)}{\dot{\gamma }}^{\left(\alpha \right)}$$

From the above basic formula of crystal deformation dynamics, the relationship between the shear stress rate and the shear strain rate on the crystal slip system, i.e., the crystal plastic constitutive relationship, can be established by further derivation. The single-crystal plastic constitutive relationship obtained from [57] is

(16) $${\hat{\sigma }}^{e}=\dot{\sigma }-W^{e}\sigma +\sigma W^{e}=C:D^{e}$$

where ${\hat{\sigma }}^{e}$ represents the Jaumann derivative of the Kirchhoff stress based on intermediate configurations, *C* represents the fourth-order elastic modulus tensor, and $\dot{\sigma }$ denotes the material derivative of the Kirchhoff stress tensor σ.

The Jaumann derivative of the Kirchhoff stress tensor based on *W* is expressed as

(17) $$\hat{\sigma }=\dot{\sigma }-W\sigma +\sigma W$$

Therefore, there is

(18) $${\hat{\sigma }}^{e}=\dot{\sigma }+W^{p}\sigma -\sigma W^{p}$$

Due to Equation (15), there is

(19) $${\hat{\sigma }}^{e}=\hat{\sigma }+\sum_{\alpha =1}^{N}B^{\left(\alpha \right)}\gamma (\alpha )$$

wherein

(20) $$B^{\left(\alpha \right)}=W^{\left(\alpha \right)}\sigma -\sigma W^{\left(\alpha \right)}$$

Simultaneously, Equations (5), (15), (16) and (18) can obtain the single-crystal constitutive equation:

(21) $$\hat{\sigma }=C:D-\sum_{\alpha =1}^{N}\left(C:P^{\left(\alpha \right)}+B^{\left(\alpha \right)}\right){\dot{\gamma }}^{\left(\alpha \right)}$$

According to Equation (21), solving the stress rate requires first solving the shear strain rate. The shear strain rates are usually calculated using rate-dependent plasticity models [59], expressed in the form of a power function as follows:

(22) $${\dot{\gamma }}^{\left(\alpha \right)}={\dot{\gamma }}_{0}{\left|\frac{\tau^{\left(\alpha \right)}}{g^{\left(\alpha \right)}}\right|}^{1/m}\mathrm{sign}(\tau^{\left(\alpha \right)})$$

where $\tau^{\left(\alpha \right)}$ denotes the component shear stress of the slip system *α*, ${\dot{\gamma }}_{0}$ denotes the reference shear strain rate, *g^(α)^* denotes the strain hardening state function, and m denotes the strain rate sensitive index. The hardening equation reflecting work-hardening behaviors adopts the Peirce–Asaro–Needleman hardening model [58]. *g^(α)^* is used to characterize the strain hardening state, assuming it is a function of the total deformation for slip *γ*,

(23) $$g^{\left(\alpha \right)}=g^{\left(\alpha \right)}(\gamma )$$

(24) $$\gamma =\sum_{\alpha =1}^{N}|\gamma^{\left(\alpha \right)}|$$

When *γ* = 0, let the initial value of *g^(α)^* be *τ_0_*:

(25) $${\dot{g}}^{\left(\alpha \right)}=\sum_{\beta =1}^{N}h_{\alpha \beta }|{\dot{\gamma }}^{\left(\alpha \right)}|$$

In the equation, *h_αβ_* represents the hardening modulus matrix:

(26) $$h_{\alpha \beta }=h_{0}[q+(1-q)\delta_{\alpha \beta }]{\mathrm{sech}}^{2}(\frac{h_{0}\gamma }{\tau_{s}-\tau_{0}})$$

where *q* denotes the coefficient describing the relative level of self-hardening and latent hardening, generally taken as 1 < *q* < 1.4; *δ_αβ_* denotes the Kronecker symbol; *h_0_* represents initial hardening rate; *τ_0_* represents yield shear stress; and *τ_s_* represents the saturation strength.

## 4. Additive Manufacturing Fatigue Simulation of Crystal Plasticity

Metal parts of additive manufacturing are often used as large integral main-load-bearing components in the aerospace field, and whether they meet the fatigue performance is the main technical indicator to measure their mechanical performance. Fatigue refers to the damage or fracture of a component under repeated action of alternating loads. The vast majority of equipment damage or failure during service is caused by fatigue.

**Figure 3:**
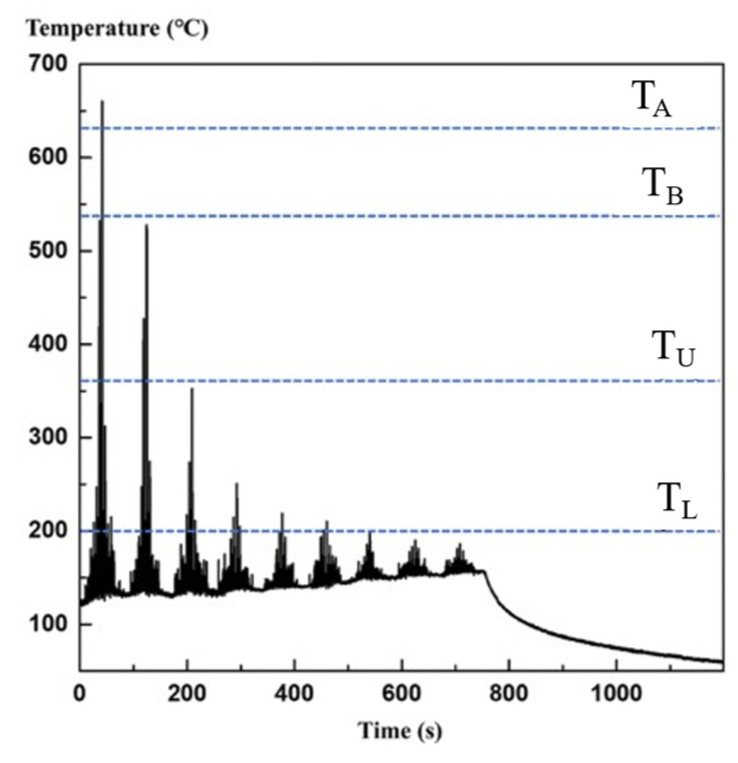
Temperature–time profile inside the molten pool during deposition process [106].

**Figure 4:**
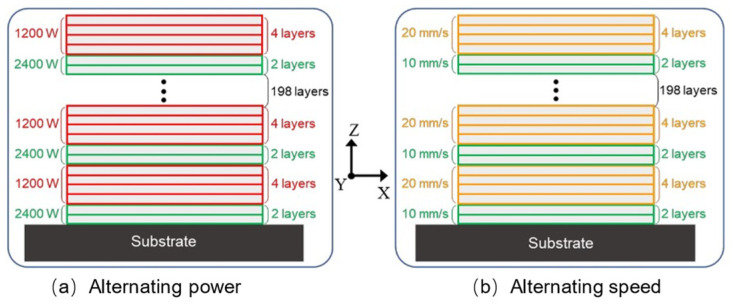
Schematic diagram of alternating forming process parameters [99].

**Figure 5:**
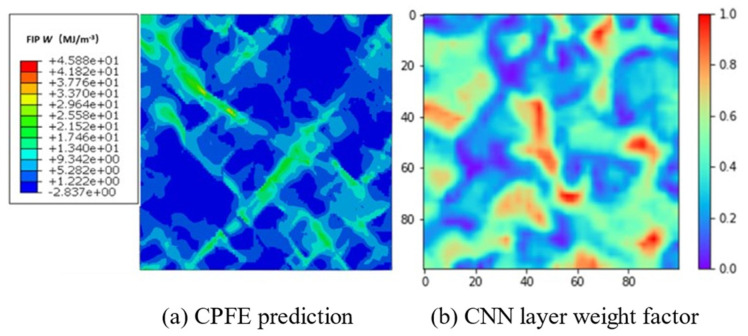
FIP W prediction contour plot [101].

Additive manufacturing heats a metal wire or powder through an energy source for melting/sintering and solidifying point-by-point, line-by-line, and surface-by-surface to form three-dimensional solid parts. Hence, the interior of the melt pool undergoes repeated thermal cycles (Figure 3). As shown in Figure 3, T_A_ represents liquidus temperature; T_B_ represents the eutectic temperature; and T_U_ and T_L_, respectively, represent the upper and lower limit temperatures that β′ precipitate. Affected by temperature gradients, columnar grains with epitaxial growth are formed through multiple cladding layers. Some scholars believe that this is the root cause of anisotropy in additive manufacturing metal materials, while others attribute it to anisotropy in the texture. The uneven microstructure causes anisotropy in the macroscopic mechanical response; therefore, the anisotropy of the microstructure is an important factor affecting the fatigue performance of additive manufacturing components. Regarding the anisotropic control strategy, from the perspective of additive manufacturing columnar grains with epitaxial growth, due to the phenomenon of columnar grains transitioning to equiaxed grains at the top of the additive manufacturing deposition layer, Zhang et al. [99] retained the equiaxed grains region (Figure 4) through the strategy of alternating the forming process parameters, blocking the growth of columnar grains, and achieved additive manufacturing of fully equiaxed grains for Ti-6Al-4V samples processed by DED. In addition, to achieve the additive manufacturing anisotropy regulation from the perspective of texture, Liu et al. [100] characterized the material texture information by EBSD, selected nine representative texture models of ODF, and simulated the Ti-6Al-4V sample produced by SLM by using the crystal plasticity finite element method, which showed that the nine textures have both positive anisotropic textures and negative anisotropic textures, and put forward the additive manufacturing metal materials anisotropy regulation strategy by combining the positive and negative anisotropic textures. Furthermore, regarding the impact of texture information on the mechanical properties of additive manufacturing, Tu et al. [101] proposed a deep learning model for strength prediction using input and output data from crystal plasticity simulations, which can accurately predict tensile properties, but the results in predicting FIP are not very reliable (Figure 5). As shown in Figure 5, compared with the crystal plasticity simulation results, the deep neural network model prediction results cannot identify the dominant grains contributed by FIP W. The use of data-driven and machine learning algorithms, as well as multi-scale and multi-physical field crystal plasticity simulations, are important research directions for achieving “structural design-fatigue performance prediction and evaluation” in additive manufacturing. Moreover, based on the advantages of crystal plasticity finite elements in micro simulations, crystal plasticity simulations of the effects of grain size and morphology [102], grain structure [103], precipitates [104], and microstructure evolution [105] on the fatigue properties of metal additive manufacturing have been reported in the literature.

**Figure 6:**
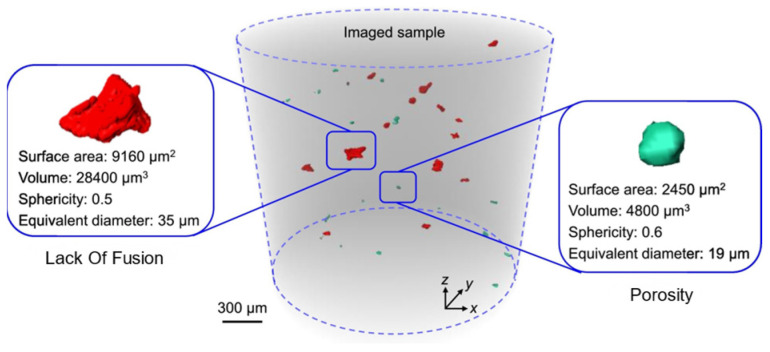
Lack of fusion and pore in synchrotron radiation X-ray imaging [111].

**Figure 7:**
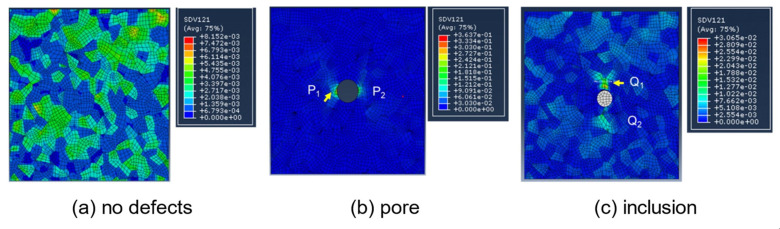
Contour of accumulated plastic shear strain [108].

**Figure 8:**
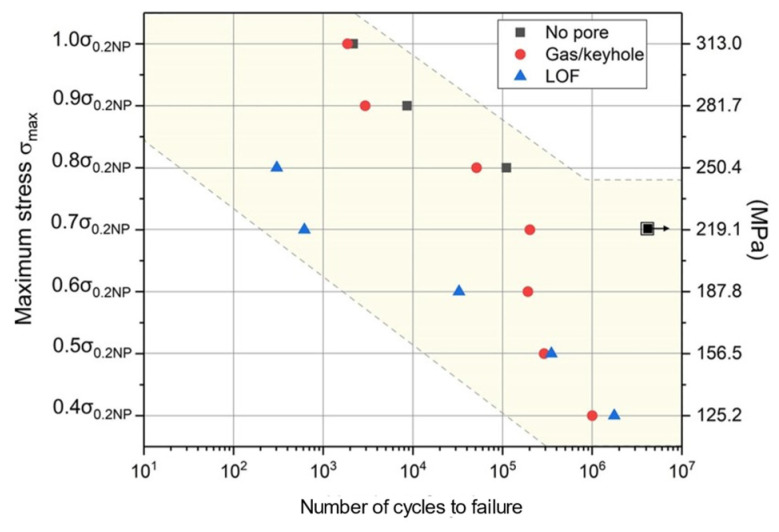
S-N data points predicted based on the storage energy density criterion [110].

The defects in metal additive manufacturing mainly include pores and lack of fusion (Figure 6): the former is due to the pores formed during the rapid solidification process of gas in the molten pool, which is relatively regular in morphology and distributed inside the molten pool, which is one of the main factors leading to the high dispersion of fatigue life of additive manufacturing components. The latter is due to poor metallurgical bonding during the material stacking process, and the defect level of lack of fusion defects can be controlled by optimizing the additive manufacturing process parameters. Manufacturing defects are the preferred nucleation sites for fatigue cracks, inducing fatigue crack initiation and greatly affecting the service reliability of components. By optimizing process parameters, hot isostatic pressing, and post-heat treatment techniques, defects in components can be reduced to a certain extent, but they cannot be completely eliminated. Additive manufacturing defects are formed by complex mechanisms and come in a variety of morphology and sizes. The type, location, size, morphology, and orientation of defects all have a significant impact on the fatigue performance of additive manufacturing components. Therefore, exploring the laws of defect formation using the above aspects can help further improve the anti-fatigue technology of additive manufacturing components and establish a fatigue life prediction model for additive manufacturing caused by defects. Reference [107] reported that accumulated plastic shear strain is associated with fatigue crack initiation. Zhang et al. [108] simulated the very-high-cycle fatigue (VHCF) behaviors of SLM-ed AlSi10Mg alloy, and it was found that the accumulated plastic shear strain near the pores and inclusions was significantly increased and was even more severe near pores (Figure 7). Luo et al. [109] simulated the high-cycle fatigue response induced by SLM-ed AlSi10Mg alloy defects and found that lack of fusion defects has a greater impact on fatigue performance compared to pores. Cao et al. [110] comparatively simulated the fatigue behaviors of SLM-ed AlSi10Mg with different pores and provided S-N curves based on the storage energy density criterion (Figure 8) to quantitatively characterize the fatigue life, as shown in the S-N curves of Figure 8. The shaded area indicates the predicted fatigue failure range, and the fatigue life of the pore and lack of fusion defects is significantly reduced, with the latter decreasing even more under high applied stress conditions. The above research indicates that, at the same scale, different types of defects present different hazards to fatigue performance.

**Figure 9:**
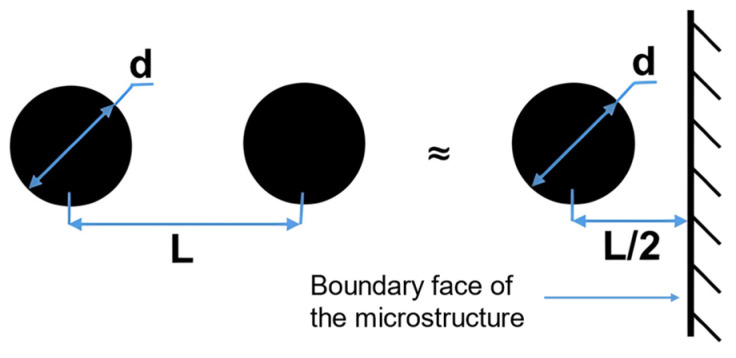
The pore-to-pore interaction problem is equated to the pore–boundary interaction [112].

**Figure 10:**
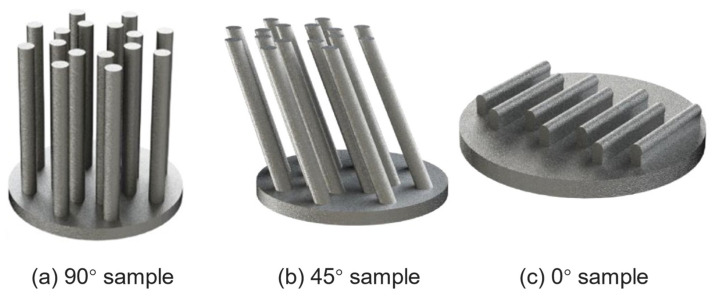
Fatigue specimens of SLM-ed AlSi10Mg alloys [113].

In addition, in terms of the influence of defect geometrical parameters (location, size, morphology, and orientation) on the fatigue performance of additive manufacturing components, Prithivirajan et al. [112] explored the critical porosity problem of additive manufacturing fatigue behaviors by taking SLM-ed IN718 as the object of study, characterizing the microstructure of SLM-ed IN718 based on EBSD. The critical porosity problem is defined in three aspects, i.e., the relative distance of pore from adjacent microstructures, the critical size of a single pore, and the distance of adjacent pores. Tthe critical porosity for fatigue failure of SLM-ed IN718 is quantitatively characterized by simulation prediction, and the pore-to-pore interaction problem is simplified and equated to the pore–boundary interaction (Figure 9). At present, the crystal plasticity simulations of geometric parameters of defects in additive manufacturing components are not yet perfect. The in-depth research on the fatigue damage mechanism of defect-induced additive manufacturing components can help to further optimize the additive manufacturing process parameters. Specifically, the additive manufacturing building direction (fatigue specimens of SLM-ed AlSi10Mg alloy with different building directions as shown in Figure 10) has a significant effect on the fatigue performance of the components. Zhang et al. [15] simulated the fatigue behaviors of SLM-ed AlSi10Mg and found that the fatigue performance of the 0° specimen was superior to that of the 90° specimen.

**Figure 11:**
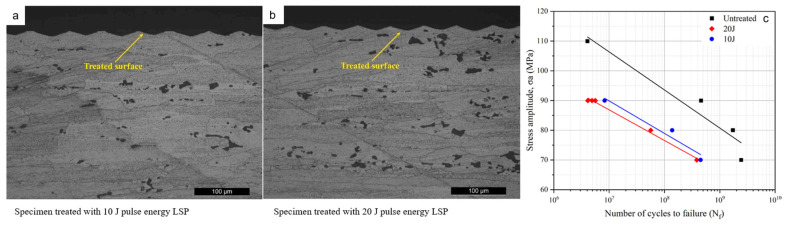
Cross-section optical microscope image and S-N curve of the LSP-treated sample [114].

Surface quality is an important factor affecting the fatigue of additive manufacturing materials. Rough-surface parts are prone to fatigue crack nucleation due to stress concentration under cyclic loading conditions. Therefore, mechanical or chemical methods are usually used to improve the surface quality. In exploring the effect of laser shot peening strengthening on the fatigue performance of aluminum alloys, Qin et al. [114] and Li et al. [115] observed through experiments that the fatigue life of 2024—t351 aluminum alloy samples gradually decreased with the increase in pulse energy levels under laser shot peening strengthening treatment (Figure 11) and explained this phenomenon by modifying the crystal plasticity finite element method. Research has found that laser shot peening leads to a decrease in very high cycle fatigue performance when considering the combined effects of grain refinement and changes in the grain aspect ratio. In addition, rapid cooling during laser movement causes a complex residual stress field inside the components, which is prone to deformation and cracking due to the effect of internal stress. Kapoor et al. [116] incorporated residual stress into the CPFE framework to study Ti-6Al-4V prepared by selective laser melting and verified the reliability of the model but did not conduct research on fatigue performance. In summary, there have been fewer applied studies of the crystal plasticity finite element method with respect to the effects of surface quality and residual stress field on fatigue performance of additive manufacturing.

In conclusion, CPFEM, as a numerical simulation tool, has been widely used to simulate the microstructure and various macroscopic mechanical responses of materials. However, most research on the fatigue performance of additive manufacturing only stays in the application stage. At the theoretical level, in terms of the characteristics of the additive manufacturing process, the introduction of metallurgical and multi-scale, multi-physical field coupling of crystal plasticity constitutive theory can more accurately characterize the fatigue behaviors of additive manufacturing materials and improve the crystal plasticity constitutive theory. In the application of the crystal plasticity finite element method, establishing a fatigue model for crystal plasticity metal additive manufacturing using data-driven and machine learning algorithms for numerical simulation can more accurately reveal the strengthening and toughening mechanism of additive manufacturing. In addition, with the help of big data, machine learning, and crystal plasticity simulation, we can establish mathematical physics equations that take into account the coupling of microstructure, defects, surface quality, residual stress, and other factors. This can be used to predict the fatigue life of metal additive manufacturing, evaluate the fatigue performance of components, realize nondestructive testing, and simultaneously be directly applied to industrial production, which can significantly reduce production costs.

## 5. Development Trends and Forecasts

Fatigue of metal additive manufacturing is a multi-scale and multi-physical field coupling problem. Crystal plasticity simulations are an important means to explore the macro–micro intrinsic relationship. This paper discusses the application of crystal plasticity simulations in additive manufacturing fatigue research from four aspects: microstructure, defects, surface quality, and residual stress. In terms of the study of fatigue performance of metal additive manufacturing, the fatigue damage mechanism is a hot and difficult point of the current research. The mechanical properties of metal additive manufacturing components, especially fatigue performance, still require further improvement. With deepening research, the development of metal additive manufacturing has shifted from early process exploration to more systematic theoretical research. A deep understanding of the microstructure characteristics of additive manufacturing metal materials can help reveal the physical essence of material strengthening and toughening. This promotes the development of high-strength and high-toughness metal additive manufacturing formation processes. At present, the following research directions need to be further strengthened.

(1) At the theoretical level, we can incorporate physical fields such as metallurgy and thermodynamics to reflect the characteristics of additive manufacturing and establish a constitutive theory of multi-physical field coupling.
(2) When applying the crystal plasticity finite element method, we can strengthen research on crystal plasticity simulation in the fatigue performance of metal additive manufacturing and explore the more essential relationship of “manufacturing process—material microstructure—fatigue performance”. In terms of the process control of additive manufacturing, exploring the optimization of microstructures, characterization of textures, defect-induced fatigue damage mechanism of metal additive manufacturing components, and other issues need more in-depth research.
(3) We can develop efficient CPFEM numerical implementation algorithms.
(4) We can develop modeling techniques that better reflect the microstructure of real materials to more accurately characterize the deformation mechanism of materials.

Numerical simulation technology is widely used. The study of metal additive manufacturing processes using crystal plasticity frameworks is of great significance for improving the mechanical properties of additive manufacturing metal components. Further research is still needed at present.

## Data Availability

Data are contained within the article.
